# Mucosal Immunization with Iron Receptor Antigens Protects against Urinary Tract Infection

**DOI:** 10.1371/journal.ppat.1000586

**Published:** 2009-09-18

**Authors:** Christopher J. Alteri, Erin C. Hagan, Kelsey E. Sivick, Sara N. Smith, Harry L. T. Mobley

**Affiliations:** Department of Microbiology and Immunology, University of Michigan Medical School, Ann Arbor, Michigan, United States of America; Northwestern University Feinberg School of Medicine, United States of America

## Abstract

Uncomplicated infections of the urinary tract, caused by uropathogenic *Escherichia coli*, are among the most common diseases requiring medical intervention. A preventive vaccine to reduce the morbidity and fiscal burden these infections have upon the healthcare system would be beneficial. Here, we describe the results of a large-scale selection process that incorporates bioinformatic, genomic, transcriptomic, and proteomic screens to identify six vaccine candidates from the 5379 predicted proteins encoded by uropathogenic *E. coli* strain CFT073. The vaccine candidates, ChuA, Hma, Iha, IreA, IroN, and IutA, all belong to a functional class of molecules that is involved in iron acquisition, a process critical for pathogenesis in all microbes. Intranasal immunization of CBA/J mice with these outer membrane iron receptors elicited a systemic and mucosal immune response that included the production of antigen-specific IgM, IgG, and IgA antibodies. The cellular response to vaccination was characterized by the induction and secretion of IFN-γ and IL-17. Of the six potential vaccine candidates, IreA, Hma, and IutA provided significant protection from experimental infection. In immunized animals, class-switching from IgM to IgG and production of antigen-specific IgA in the urine represent immunological correlates of protection from *E. coli* bladder colonization. These findings are an important first step toward the development of a subunit vaccine to prevent urinary tract infections and demonstrate how targeting an entire class of molecules that are collectively required for pathogenesis may represent a fundamental strategy to combat infections.

## Author Summary

Because urinary tract infections (UTIs) are a significant healthcare burden, it would be beneficial to develop a vaccine to prevent uncomplicated UTI caused by *Escherichia coli*. Using a large-scale screening process we uniformly identified proteins involved in iron uptake as potential vaccine candidates against *E. coli*. Iron acquisition is a critical function required by bacteria in order to cause infections. In uropathogenic *Escherichia coli*, this function is mediated by a repertoire of systems that scavenge iron from the host during infection. We found that vaccination with certain iron receptors from these systems is sufficient to elicit protective immunity from experimental urinary tract infection. Induction of an antibody response played a key role in protection from infection because antibody class-switching and the production of antibodies in urine correlated with reduced numbers of bacteria in the bladder. By targeting an entire class of molecules involved in iron acquisition instead of a single protein, it was possible to successfully identify components of a protective UTI vaccine. This strategy could be a useful approach in the development of vaccines to prevent infections caused by other pathogenic bacteria.

## Introduction

The urinary tract is among the most common sites of bacterial infection. Over half (53%) of all women and 14% of men experience at least one urinary tract infection (UTI) in their lifetime [1],[2], leading to an average of 6.8 million physician office visits, 1.3 million emergency room visits, and 245,000 hospitalizations per year, with an annual cost of over $2.4 billion in the United States alone [3]. *Escherichia coli* is the infectious agent in more than 80% of uncomplicated UTIs, which occur in patients with an anatomically normal urinary tract devoid of structural abnormalities or inflammatory lesions [4].

In addition to symptoms of acute cystitis and pyelonephritis caused by UTI, a number of more serious conditions are often associated with these infections. Upper UTIs in young children can cause permanent kidney damage. An estimated 57% of children with acute pyelonephritis develop renal scarring [5]. UTIs are classically treated with trimethoprim/sulfamethoxazole or ciprofloxacin to eradicate the infecting strain. However, there is documentation of increasing resistance to these antibiotics [6]. Furthermore, following successful primary treatment, recurrent infections frequently occur, with an estimated 27% of women experiencing a recurrence within six months of the original infection and 2.7% experiencing a third infection during this time [7]. The reservoir for reinfection remains unclear, with same-strain episodes making up between 25–100% of recurrent UTI cases [reviewed in [8]]. Consequently, these complications pose a significant challenge to UTI treatment and suggest that a vaccine to prevent UTI would alleviate this major source of morbidity and economic burden.

Indeed, a number of groups have sought to stimulate protective immunity against UPEC. Early studies utilized various capsular and LPS core antigens and heat-killed bacteria to elicit protective immune responses [9],[10]. Recently, whole cell or cell extract preparations have been shown to provide modest short-term protection in some individuals [11],[12]. Because of their abundance on the cell surface and demonstrated role in UPEC pathogenesis [13], fimbriae have been attractive targets for defined protein subunit vaccines. For example, immunization with the type 1 fimbrial adhesin, FimH, conjugated to its periplasmic chaperone, FimC, reduced murine bladder colonization by 99.9%, as well as provided protection in a primate model [14],[15]. Additionally, subunit vaccines based on several other surface-exposed molecules such as P fimbriae (PapDG complex), alpha hemolysin, Dr fimbriae, the salmochelin receptor IroN, and a conjugation of capsule polysaccharide K13 with diphtheria toxoid have been shown to induce at least some immune response in immunized animals [16]–[20]. However, although much research has focused on the development of a vaccine against UPEC, none are available in the United States.

Large-scale reverse vaccinology approaches offer an alternative to traditional vaccine design. Pioneered by successful work using *Neisseria meningitidis*, this technique applies genomic and bioinformatic methods to identify novel vaccine targets [21]. Recently applied to extraintestinal pathogenic *E. coli* (ExPEC), a pathotype to which UPEC belongs, a subtractive hybridization study identified surface-exposed antigens specific to ExPEC and found that several of these proteins protected immunized mice from lethal sepsis [22]. Due to the limited success of previous UTI vaccine design strategies, we hypothesized that a functional vaccinology approach would identify vaccine targets of UPEC in an unbiased manner that could elicit protective immunity.

Here we describe the use of previously established genomics and proteomics data to identify six pathogen-associated outer membrane iron receptors (ChuA, Hma, Iha, IreA, IroN and IutA) as putative vaccine targets of UPEC. Each of these 71–84 kDa proteins is predicted to form a transmembrane beta-barrel in the outer membrane, with a series of loops extending extracellularly [23]. Facilitating import of specific iron sources, these receptors mediate uptake of siderophores, secreted bacterial iron-chelating molecules, or host heme-derived iron. Because iron acquisition is necessary for bacterial pathogenesis and it is well known that the urinary tract is an iron-limited environment, iron acquisition via these receptors is crucial for UPEC infection [24]. Consequently, deletion of the siderophore receptor IreA, heme receptors ChuA or Hma, enterobactin receptor Iha, salmochelin receptor IroN, or aerobactin receptor IutA all decrease the fitness of UPEC in the murine urinary tract [24]–[28].

In this study, we demonstrate that an unbiased, rational vaccinology approach consistently identified a class of molecules involved in iron acquisition as vaccine candidates and that intranasal immunization with these UPEC outer membrane iron receptors provides protection from UTI. Additionally, we show that antigen-specific antibody and cytokine responses are generated in response to vaccination with iron receptor proteins, of which, the former correlated with protection. Therefore, we propose that this class of molecules is promising as protective vaccine targets against UPEC and, because of their conserved function of iron acquisition for pathogenesis, could potentially be adopted for the development of vaccines against other Gram-negative bacterial infections.

## Results

### Candidate antigen selection

To identify bacterial proteins that could be used as vaccine targets against UPEC infection, we utilized a functional vaccinology approach, combining both genomics and proteomics techniques. To begin, criteria defining UPEC vaccine targets were established and data from our previously-described studies assembled to identify proteins meeting these requirements. Of the 5379 predicted proteins in *E. coli* pyelonephritis strain CFT073, only six proteins (Table 1) met all five of our established criteria: high *in vivo* expression, induction during growth in human urine, surface exposure, antigenicity, and pathogen-specificity.

**Table 1:** Candidate antigens and selection criteria.

Gene	Locus tag	Fold change *in vivo* a	*in vivo* percentile^b^	Fold change in human urine^c^	Antigenic^d^	% UPEC (*n* = 55)^e^	% fecal (*n* = 30)^e^
*chuA*	c4308	7.06	95.0	27.8	+	87	30
*hma*	c2482	6.56	94.5	14.8	+	69	17
*iha*	c3610	18.9	89.1	5.87	+	45	37
*ireA*	c5174	23.3	95.2	7.81	+	20	17
*iroN*	c1250	22.7	82.7	7.63	+	71	33
*iutA*	c3623	5.57	97.0	49.2	+	65	17

a Transcript fold change in the urine of experimentally infected mice as compared to growth in LB [29].

b Of 5379 genes ranked in order of *in vivo* transcript microarray signal intensity [29].

c Protein fold change following growth in pooled filter-sterilized human urine as compared to growth in LB [30].

d Reacts with sera from mice chronically infected with *E. coli* CFT073 [31].

e Prevalence of indicated gene in UPEC and fecal isolates [31].

Transcriptomic and proteomic data were evaluated to identify candidates meeting the expression and antigenicity criteria. Data from an *in vivo* transcriptome study indicated that genes encoding iron acquisition system components were among those most highly upregulated in CFT073 isolated from the urine of experimentally infected CBA/J mice [29]. When all genes were ranked in order of expression level *in vivo*, these iron acquisition genes, specifically outer membrane iron receptors, were among the top 18% most highly expressed in the murine urinary tract (Table 1). Similarly, when outer membrane proteins (OMPs), which are partially surface-exposed, were isolated from CFT073 cultured in human urine *ex vivo* and compared with OMPs isolated from bacteria cultured in Luria broth, these iron receptors were the most highly induced proteins [30]. Further, the vaccine candidates listed in Table 1 comprised six of the top seven human urine-induced OMPs. In addition to bacteria isolated from urine, our and other groups have observed expression of these outer membrane iron receptors by bladder cell-associated UPEC, both *in vitro* [31] and *in vivo* [32]. Finally, an immunoproteomics study identified these iron receptors as antigenic; that is, they reacted with antisera from mice chronically infected with UPEC strain CFT073, indicating that these proteins elicited a humoral response during experimental UTI [31].

As UPEC strains represent a subset of strains that are genetically distinct from commensal *E. coli*, a vaccine directed against UPEC should specifically target pathogenic strains. Toward this end, a comparative genomics hybridization study identified 131 genes that were present in all UPEC strains analyzed (*n* = 10), but none of the fecal-commensal *E. coli* isolates tested (*n* = 4) [33]. Among these UPEC-specific genes were two encoding outer membrane heme receptors, *chuA* and *hma*. Furthermore, dot blot analysis of a collection of UPEC and fecal-commensal *E. coli* isolates identified several of these iron OMP genes (*chuA*, *hma*, *iroN*, and *iutA*) as present more frequently among pathogens than non-pathogens [31]. Even genes that were not statistically more frequent among uropathogens (*iha* and *ireA*) were nonetheless present at relatively low frequencies in commensals.

Of the 5379 predicted proteins in UPEC strain CFT073, six candidate antigens, all outer membrane iron receptors, emerged from our series of genomics and proteomics studies as uniformly highly ranked potential vaccine targets (Table 1). This screening process strongly suggested that broadly targeting an entire class of molecules involved in iron acquisition could be an effective strategy to develop a protective UTI vaccine.

### Vaccination confers protection against experimental UTI

The six iron receptor vaccine candidates, ChuA, Hma, IutA, IreA, Iha, and IroN were expressed and purified as affinity-tagged recombinant proteins. Consistent with the predicted structure of these antigens, the CD spectrum of refolded purified Hma displayed a trough at 218 nm, which is characteristic of a β-sheet-rich conformation (Fig. S1). The six purified protein antigens were each biochemically cross-linked to the adjuvant cholera toxin (CT) at a ratio of 10∶1 (antigen∶CT) and groups of mice (*n* = 15–20) were intranasally inoculated with either an antigen-CT complex or CT alone. Following primary immunization (day 0) and booster doses (days 7 and 14), the animals were transurethrally challenged with UPEC strain CFT073 and protection was assessed at 48 h post infection (hpi) by determining CFUs in the urine, bladder, and kidneys.

**Figure 1:**
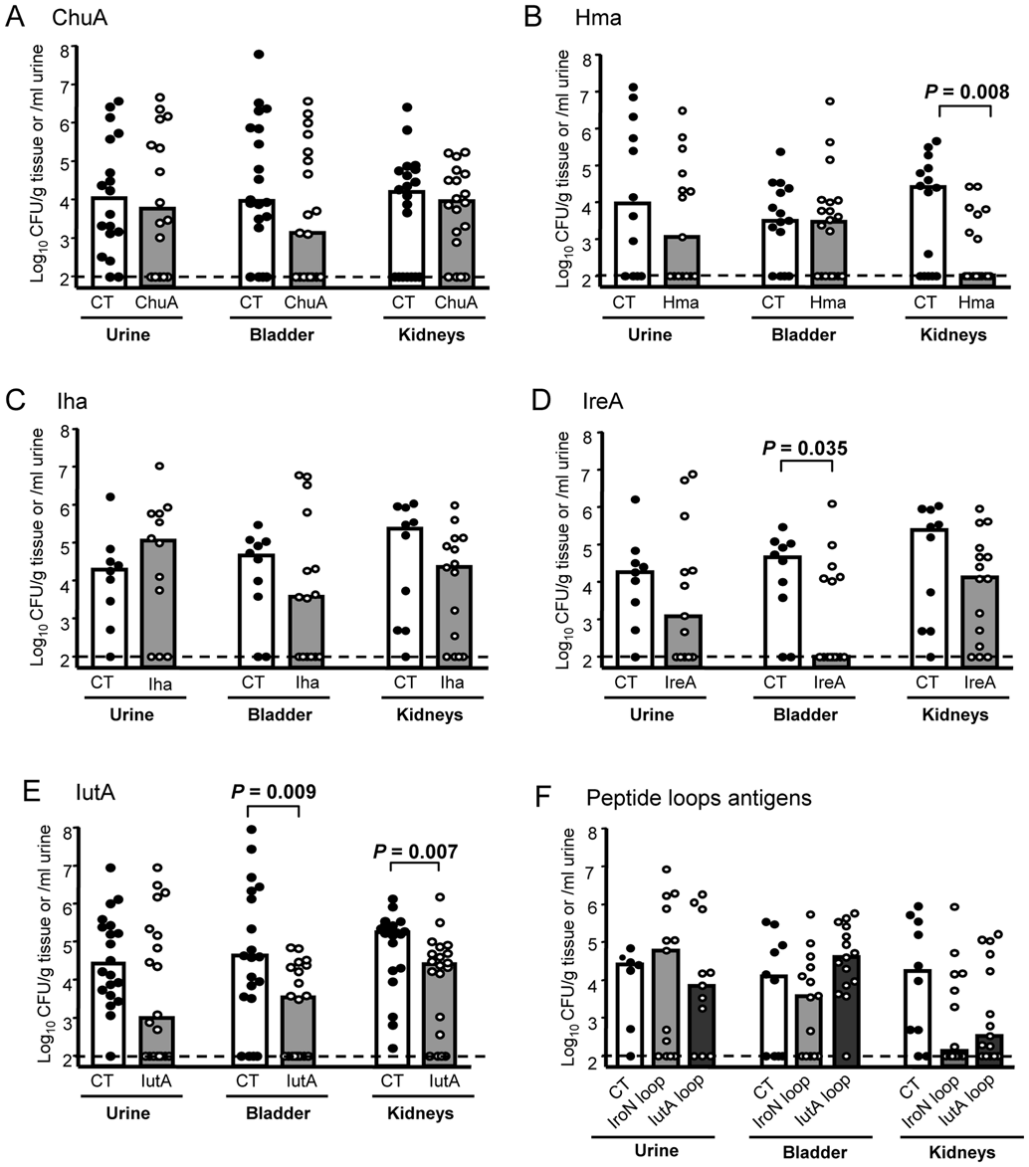
Immunization with outer membrane iron receptor antigens protects against urinary tract colonization by *E. coli* CFT073. CBA/J mice were intranasally vaccinated as described with a primary dose of 100 µg purified protein crosslinked to 10 µg CT (A–E) or 100 µg peptide mixed with 10 µg CT (F), followed by 2 boosts of 25 µg antigen crosslinked to 2.5 µg CT or mixed with CT for peptides. One week following the final boost, animals were transurethrally challenged with 1×10^8^ CFU of *E. coli* CFT073 and colonization was measured 48 hpi. Symbols represent CFU/g tissue or /ml urine of individual mice and bars indicate median values. Clear bars, mock vaccinated with CT alone; gray bars, vaccinated with purified (A) ChuA, (B) Hma, (C) Iha, (D) IreA, or (E) IutA, or (F) peptides IroN (light gray bars) or IutA (dark gray bars). Dashed line shows the limit of detection for this assay, 100 CFU/g. Significance was determined using a one-tailed Mann-Whitney test.

Of the six candidates, three conferred protection against experimental challenge with UPEC. The heme receptor, Hma, protected mice against colonization of the kidney. Hma-vaccinated mice demonstrated nearly a 3-log reduction in median CFU/g in the kidney (*P* = 0.008) and 13/20 mice had undetectable levels of bacteria (<100 CFU/g) in the kidneys (Fig. 1B). The putative siderophore receptor IreA showed significant protection and demonstrated a 3-log reduction in median CFU/g in the bladder (*P* = 0.035) (Fig. 1D). For IreA, 9/15 vaccinated mice had undetectable levels of bacteria in the bladder. The siderophore receptor for aerobactin, IutA, conferred significant protection against UPEC challenge in both the bladder and kidneys. Mice vaccinated with IutA displayed one-log CFU/g reduction in both the bladder (*P* = 0.009) and kidneys (*P* = 0.007) (Fig. 1E). This showed that mucosal immunization in the nares generates a protective effect at distal sites, the bladder and kidneys. Not all antigens selected as candidates provided protection against experimental UTI. Both ChuA and Iha recombinant proteins when cross-linked to cholera toxin failed to elicit significant protection against challenge with UPEC strain CFT073 (Fig. 1A and C). All of the native protein vaccines were well tolerated in immunized mice and no animals died following vaccination, with the exception of ChuA, which was lethal in 11/30 mice (surviving mice were not protected).

**Figure 2:**
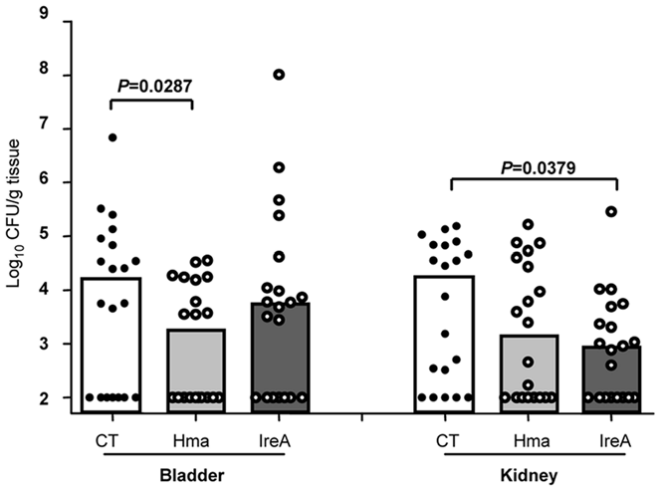
Immunization with Hma and IreA reduces colonization by *E. coli* 536. CBA/J mice were intranasally vaccinated as described with a primary dose of 100 µg purified protein crosslinked to 10 µg CT (open circles) or CT alone (closed circles), followed by 2 boosts of 25 µg antigen crosslinked to 2.5 µg CT or CT alone. One week following the final boost, animals were transurethrally challenged with 1×10^8^ CFU of *E. coli* 536 and colonization was measured 48 hpi. Symbols represent CFU/g tissue or /ml urine of individual mice and bars indicate median values. Clear bars, mock vaccinated with CT alone; gray bars, vaccinated with purified Hma or IreA. Significance was determined using a one-tailed Mann-Whitney test.

Since a significant reduction in post-challenge CFU was observed for Hma and IreA vaccinated mice, these antigens were tested to determine if similar levels of protection could be generated following heterologous challenge with another UPEC isolate. Groups of 20 mice were immunized as described with either Hma, IreA, or CT alone and CFU were determined at 48 hpi following challenge with UPEC strain 536. In these experiments, Hma vaccinated mice were significantly protected from heterologous challenge and displayed >10-fold reduction in median CFU/g in the bladder (*P* = 0.0287) (Fig. 2). Vaccination with IreA significantly protected mice from infection with UPEC strain 536 and these mice demonstrated a log-fold decrease in median CFU/g in the kidneys (*P* = 0.0379) (Fig. 2).

Peptides (30 aa) corresponding to single predicted extracellular loops in both the salmochelin siderophore receptor IroN (aa 491–520) and aerobactin receptor IutA (aa 467–498) were also assessed to determine their use as potential immunogens. Extracellular loops were selected based on their predicted topology to direct an antibody response against surface exposed residues. These peptides also represent highly conserved loops within the salmochelin and aerobactin receptors; the IroN peptide has >90% identity to 37 siderophore receptor sequences including urinary tract isolates 536, UTI89, and 83972. The IutA peptide has >76% identity (23/30 aa) among 15 aerobactin receptor sequences present in various pathogenic *E. coli*, including uropathogenic and extraintestinal isolates UMN026 [34], IAI39, and S88 [35]. Groups of mice (*n* = 15) were inoculated as previously described with either peptide mixed with CT or CT alone and protection was assessed at 48 hpi following infection with UPEC strain CFT073. These peptides failed to elicit significant protection in the urine, bladder, or kidneys of mice, however, there was nearly a 2-log reduction in CFU/g in the kidneys for both IroN (*P* = 0.053) and IutA (*P* = 0.078) peptides, demonstrating a strong trend towards protection (Fig. 1F). These results imply that peptides may be suitable for development of a UTI vaccine but may be more efficacious if cross-linked or fused to a carrier molecule. Together, these findings show that targeting an entire functional class of molecules involved in iron acquisition is an effective strategy to identify protective vaccine candidates that significantly reduce bacterial colonization during ascending UTI following experimental challenge with UPEC.

### Antigen-specific splenocytes from vaccinated mice secrete IFN-γ and IL-17

**Figure 3:**
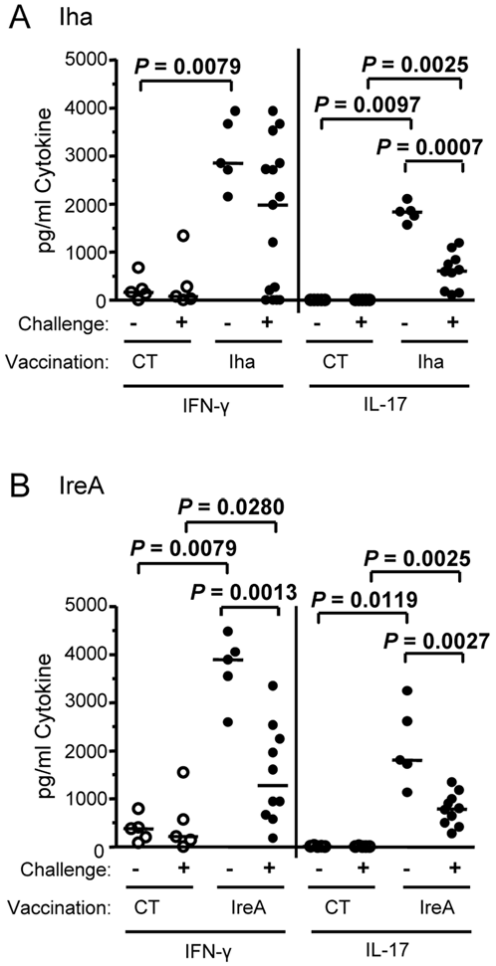
Antigen-specific splenocytes from vaccinated mice secrete IFN-γ and IL-17. Splenocytes harvested from (A) Iha-vaccinated or (B) IreA-vaccinated without challenge (−) or two days after transurethral challenge with *E. coli* CFT073 (+) were stimulated with the corresponding antigen *in vitro*. Cytokines were measured in culture supernatants by ELISA. Open circles represent CT control-treated mice and closed circles represent iron receptor antigen-vaccinated mice. Each circle represents an individual animal and bars represent the median. Significance was determined using a two-tailed Mann-Whitney test.

Proinflammatory cytokines are crucial for orchestrating protective immune responses against pathogens. Secretion of two major proinflammatory cytokine mediators, IFN-γ and IL-17A (IL-17), were measured from the splenocytes of mice immunized with adjuvant alone (CT), IreA (a protective antigen), or Iha (a non-protective antigen) following vaccination. Cells were analyzed both pre- and post-challenge to gauge the immune response to antigenic stimulation *in vivo*. Splenocytes were cultured *in vitro* in the presence of 1 µg/ml of the corresponding purified antigen used for immunization. Cytokines were measured in the supernatant by ELISA after 48 h incubation. Splenocytes from Iha-vaccinated and IreA-vaccinated mice collected both before (−) and after (+) challenge demonstrated significant antigen-specific secretion of IFN-γ and IL-17 when compared to the corresponding splenocytes from CT control mice (*P*<0.05) (Fig. 3A and B). Unstimulated splenocytes from any group of mice lacked detectable levels of IFN-γ or IL-17 (data not shown). Interestingly, splenocytes derived from Iha- and IreA-vaccinated mice post-challenge secreted less pro-inflammatory cytokines than pre-challenge cells (*P*<0.003) (Fig. 3A and B). Both IFN-γ and IL-17 secreting splenocytes were significantly decreased post-challenge (*P*<0.003) in IreA-vaccinated mice (Fig 3B), whereas only IL-17 secreted splenocytes were significantly decreased (*P* = 0.0007) in mice vaccinated with Iha (Fig. 3A). Overall, these results indicated that vaccinated mice generate antigen-specific cells that are capable of secreting IFN-γ and IL-17, two important pro-inflammatory cytokine mediators, in response to *in vitro* restimulation. No significant correlation was found between cytokine production and reduction in CFU post-challenge. However, the decrease in both IFN-γ and IL-17 secretion from vaccinated mice post-challenge as compared to pre-challenge was only observed following immunization with antigens that protected mice from infection (Fig. 3B) and was not seen following immunization with non-protective antigens (Fig. 3A).

### Vaccinated mice secrete antigen-specific IgA in urine

**Figure 4:**
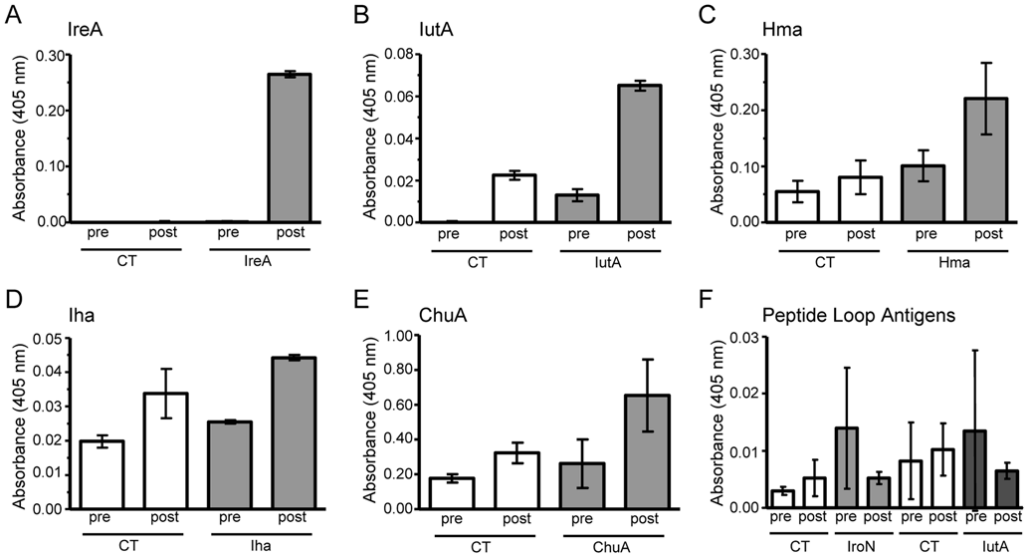
Mice produce antigen-specific urinary IgA in response to intranasal immunization. Urine from (A) IreA-vaccinated, (B) IutA-vaccinated, (C) Hma-vaccinated, (D) Iha-vaccinated, (E) ChuA-vaccinated, (F) peptide-vaccinated, and corresponding CT control mice was collected prior to vaccination (pre) and after vaccination but before transurethral challenge with UPEC strain CFT073 (post). Pooled samples were plated in appropriately coated ELISA plates and probed for antigen-specific IgA. Open bars represent CT control mice and shaded bars represent values from vaccinated mice. Absorbance reflects relative quantity of IgA. Error bars indicate the mean+/−SEM of two experiments.

To evaluate the humoral immune response at the primary site of infection, the bladder mucosa, levels of antigen-specific IgA in urine were measured by ELISA. To account for variability in the amount and concentration of urine from each mouse, collections were pooled for use in an indirect ELISA to measure antigen-specific IgA. Urine from IreA- (Fig. 4A) and IutA-vaccinated (Fig. 4B) cohorts, which had significantly decreased bladder colonization upon challenge, had the highest fold increases in IgA (34-fold and 6.2-fold, respectively) post-vaccination. Antigens that did not generate significant decreases in colonization in the bladders of vaccinated mice had more modest fold-increases in urine IgA post-vaccination (2.2-fold for Hma, 2.0-fold for ChuA, and 1.7-fold for Iha) (Fig. 4C–E). Correspondingly, peptide antigens representing extracellular loops of IroN and IutA did not significantly protect from infection in the bladder, and IgA was at background levels in the urine of these animals (Fig. 4F). These findings demonstrate that vaccination with antigens, IreA and IutA, which provide significant protection in the bladder from experimental challenge with UPEC, generate significant induction of antigen-specific IgA secretion that is detectable in urine of immunized animals.

### Vaccinated mice produce antigen-specific serum antibodies

**Figure 5:**
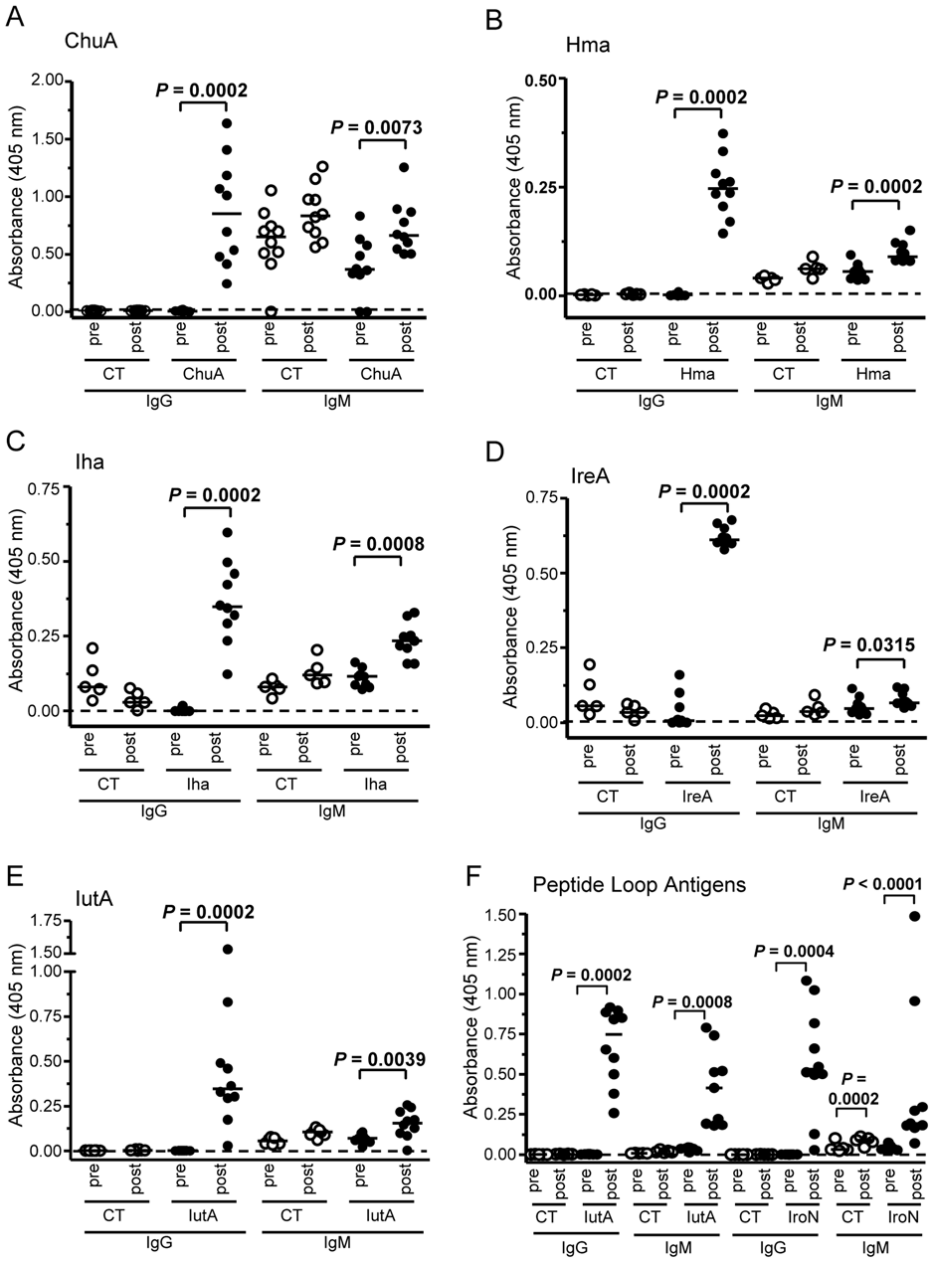
Antigen-specific serum IgM and IgG is produced in vaccinated mice. Serum from (A) ChuA-vaccinated, (B) Hma-vaccinated, (C) Iha-vaccinated, (D) IreA-vaccinated, (E) IutA-vaccinated, (F) IroN and IutA extracellular loop peptide antigens and corresponding CT control-treated mice was collected prior to vaccination (pre) and after vaccination before transurethral challenge with UPEC strain CFT073 (post). Samples were plated in duplicate in appropriately coated ELISA plates and probed for relative IgG and IgM. Open circles represent CT control mice and closed circles represent vaccinated mice. Each circle represents an individual animal and bars represent the median. Significance was determined using a two-tailed Mann-Whitney test.

To evaluate the systemic humoral immune response to our vaccine candidates, the levels of serum antigen-specific antibodies generated by vaccinated mice were compared to those of CT control mice. Serum was collected by infraorbital ocular bleed before vaccination and after vaccination prior to transurethral challenge with UPEC strain CFT073. For each protein and loop peptide antigen tested, there was a significant increase of antigen-specific IgG in post-vaccination serum compared to the corresponding pre-immune serum (*P* = 0.0002) (Fig. 5A–F). In most cases, there was no difference in IgG levels between the pre- and post-vaccinated sera from CT control mice. Additionally, all post-vaccination sera had significantly higher levels of IgM than pre-vaccination sera (*P*<0.05) (Fig. 5A–F). Although both IgG1 and IgG2a increased from pre- to post-vaccination, there was no skew toward production of IgG1 or IgG2a in any group (data not shown). This phenomenon is not unusual for protein antigens, as they can stimulate production of both Th1 and Th2 cells by virtue of their broad MHC class II peptide repertoire [36]. None of the samples tested showed appreciable increases in serum IgA (data not shown). These findings show that vaccinated mice generate target-specific antibodies, both innate IgM and class-switched IgG in response to intranasal immunization with iron-receptor antigens.

### Antibody class-switching and mucosal IgA correlates with protection

**Figure 6:**
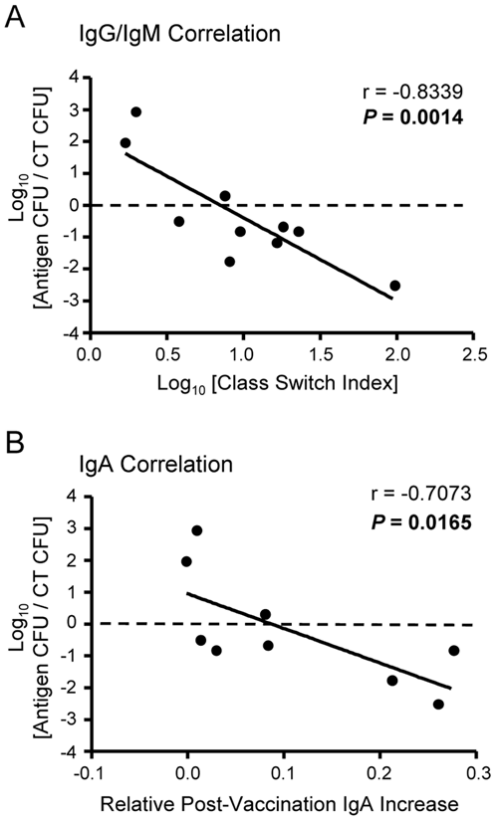
Isotype class-switching and urinary IgA correlates with vaccine-mediated protection from infection. (A) The Class Switch Index was calculated by dividing the change in serum IgG absorbance for the entire group of vaccinated mice (accounting for non-specific increases by subtracting the corresponding median change in IgG absorbance for the CT-vaccinated mouse cohort) by the same computation preformed with group median IgM absorbance values [(ΔIgG_VAC_−ΔIgG_CT_)/(ΔIgM_VAC_−ΔIgM_CT_)]. The Class Switch Index was then plotted against the normalized bladder CFU (group median CFU_VAC_/group median CFU_CT_) for each vaccination experiment. Thus, each individual point reflects the collective data from an experiment involving 10 vaccinated and 10 CT mice (*n* = 203). Lower normalized CFU values indicate enhanced bacterial clearance after challenge, where higher class switch indices represent superior B cell activation by comparing the increase in IgG compared to the increase in IgM. (B) Normalized bladder CFU values from each vaccination experiment are plotted against the median change in absorbance of antigen-specfic IgA detected in the urine (ΔIgA_VAC_). Pearson correlation coefficient (*r*) and *P* values are shown.

IgG and IgM are often monitored to determine the level of class switching undergone by B cells. In our studies, all vaccinated animals demonstrated significant increases in both serum IgG and IgM from pre- to post-vaccination. However, Hma-, IreA-, and IutA-vaccinated (protected) animals displayed more dramatic IgG increases than IgM when compared to ChuA-, Iha-, and peptide loop antigen-vaccinated (unprotected) animals (compare Fig. 5B, D, and E to Fig. 5A, C, and F). This finding suggested that these animals have been sufficiently stimulated to class switch to more effective antibody isotypes. To more quantitatively assess this trend and the relationship of serum IgG and IgM to protection from UTI, we first calculated a Class Switch Index using the data from all of the immunized mice (*n* = 203). The Class Switch Index is the ratio of the median change in sera IgG to the median change in sera IgM for each group of vaccinated mice. To account for an increase in non-specific antibodies, the median changes in sera IgG and IgM of CT-treated mouse cohorts were subtracted from the corresponding antigen-vaccinated group values. The class switch indices were then plotted against the normalized post-challenge median bladder CFU/g to determine if any relationship existed between antibody class-switching and protection from cystitis. Because infectivity can vary between experiments, the median CFU/g from each antigen-vaccinated group was divided by the median CFU/g of the CT control group for normalization. Finally, both the Class Switch Index and normalized median CFU/g values were log_10_ transformed for comparison on a linear scale. This analysis demonstrated that there was a significant correlation between the Class Switch Index and the CFU/g in the bladder (*P* = 0.0014) (Fig. 6A), suggesting that a decrease in bladder colonization may be attributed to the relative amount of antibody class-switching from IgM to IgG in mice vaccinated with protective antigens. No significant correlation was found between antibody class-switching from IgM to IgG and CFU/g in the kidneys. However, as predicted, there is also a strong correlation between the amount of antigen-specific IgA in urine and protection from UPEC infection in the bladder (Fig. 6B); specifically, increases in urine IgA corresponded to decreases in bladder CFU (*P* = 0.0165). These findings show that antibody class-switching and urinary IgA are each an immunological correlate of protection against UTI caused by *E. coli*.

## Discussion

UTIs caused by UPEC represent a significant healthcare burden that could be alleviated by the development of a vaccine that would provide protection from these infections. Toward this aim, we described the use of a multi-pronged, functional vaccinology approach that uniformly singled out one class of molecules, those involved in iron acquisition, as potential targets. The screening process selected six vaccine candidates from the 5379 predicted proteins encoded within the *E. coli* CFT073 genome. The six vaccine candidates meet all of the following criteria: predicted to be surface-exposed, present in the bacterial outer membrane [30],[31], conserved among UPEC strains [31],[33], transcriptionally upregulated *in vivo* [29], induced during culture in human urine [30], and antigenic [31]. These vaccine targets, ChuA, Hma, Iha, IreA, IroN, and IutA, are all outer membrane β-barrel proteins that function as receptors for iron-containing compounds. Of these, we found that intranasal immunization with Hma, IreA, or IutA generates an antigen-specific humoral response, antigen-specific production of IL-17 and IFN-γ, and provides significant protection against experimental infection with UPEC.

Site-specific protection was observed for the antigens that provided the greatest reduction in bacterial counts, Hma and IreA. Mice immunized with the IreA vaccine were significantly protected from CFT073 colonization only in the bladder, with >2-log reduction in the median CFU/g and 60% of mice having undetectable levels of bacteria within that organ. The dramatic reduction in the level of bacterial colonization within the bladder may have resulted in reduced numbers of bacteria ascending to the kidneys that could account for the modest reduction in bacteria seen in the kidneys in the IreA-vaccinated mice following challenge with either UPEC strain CFT073 or 536.

In contrast to the bladder protection seen with IreA, Hma significantly reduced the number of bacteria colonizing the kidneys and, in over half of the animals, prevented kidney colonization completely. Despite the significant protection in the kidney, immunization with the Hma vaccine did not reduce the level of bacteria able to colonize the bladder. The kidney-specific protection of the Hma vaccine may reflect the biological function of Hma for UPEC during colonization of the urinary tract, UPEC that are unable to produce Hma have reduced fitness only within the kidney during experimental UTI [28]. The site-specificity for the Hma vaccines suggests that the immune response may perturb the normal function of this outer membrane heme receptor, perhaps by antibody interference with ligand-binding domains or selective immune targeting of bacteria that exhibit tissue-specific expression of Hma.

Induction of the cytokines IL-17 and IFN-γ suggests that the cellular response may be important for the generation of a protective immune response. To assess cellular responses to immunization, we analyzed antigen-specific production of two cytokines known to be critical for mediating anti-bacterial activities within the host, IFN-γ and IL-17, from splenocytes post-immunization both before and after challenge. Production of IFN- γ has been previously shown to be important for the control of infection within the urinary tract [37] and IL-17 has been shown to promote the recruitment of neutrophils in response to bacterial infection [38]. IL-17 has been recently appreciated as an important mediator to control infection by *Salmonella*, *Listeria*, pathogenic *Mycobacterium*, *E. coli*, and *Klebsiella* [39]–[43]. Both IFN- γ and IL-17 were produced in response to immunization with IreA and Iha. Splenocytes obtained from vaccinated mice post-challenge showed a reduction in antigen-specific cytokine secretion of IFN- γ and IL-17, suggesting that antigen-specific lymphocytes may be homing from the spleen to the site of infection. Further, the post-challenge reduction in both antigen-specific cytokines tested was only significant for the IreA vaccine, which generates a protective response in the bladder.

Mucosal immunization is considered the most effective means to develop a UTI vaccine and we have shown previously that intranasal immunization with MR/P fimbria is effective in protecting mice against UTI caused by *Proteus mirabilis*, an agent of complicated UTI [44]. Because local immunization of the urethra is not practical, the migration of immune cells between mucosal sites can be exploited during intranasal inoculation with antigen [45]. We reasoned that intranasal immunization would generate a distant mucosal response in the genitourinary tract against UPEC in vaccinated animals. Consistent with this, we observed that the two vaccines, IreA and IutA, which conferred significant protection in the bladder, had the greatest antigen-specific production of secretory IgA detectable in their urine. For the IreA vaccine, the increase in IgA in urine provides evidence that may account for the bladder-specific protection seen in mice immunized with this vaccine. Conversely, antigens that did not induce protective immunity within the bladder did not generate a similar increase in the level of IgA. Based upon these findings, relative levels of IgA in the urine significantly correlated with protection in the bladder. That is, when examining all of the vaccine candidates and bacterial counts within the bladder, there is a direct relationship between a reduction in CFU in the bladder and increased IgA in the urine. This immunological correlate suggests that a mucosal response and vaccine that generates IgA is sufficient to provide protection from UPEC colonization in the bladder. This finding is consistent with other studies that have shown that antibodies specific for the infecting *E. coli* strains leads to resolution of cystitis and that oral immunization with outer membrane proteins generates an antigen-specific IgA mucosal response against UPEC [46],[47].

The finding that IgA in the urine is produced in response to intranasal immunization indirectly shows that class-switching of antibody isotypes is occurring in vaccinated animals. Analysis of serum antibodies from immunized mice shows that antigen-specific IgM is produced in response to all the vaccine candidates tested. Further, antigen-specific IgG is markedly increased in response to each vaccine when compared to pre-immune sera. For the Hma and IutA vaccines, which provided significant protection in the kidney, it is possible that circulating IgG or antigen-specific plasma cells contribute to the observed reduction of bacterial colonization. Interestingly, the vaccines that significantly protected mice against UPEC infection, Hma, IreA, and IutA, displayed a dramatic increase in antigen-specific IgG relative to IgM. This suggests that class-switching of antibody isotypes is indicative of a protective immune response. To assess this effect, we defined a Class Switch Index by comparing the ratio derived from the median changes in antigen-specific IgG to that of IgM, normalized to CT and plotted those values against the CT-normalized log-ratio of CFU/g in the bladder post-challenge. By plotting normalized CFU/g values against the Class Switch Index, we found a significant correlation between class-switching antibody isotypes and protection from bacterial colonization in the bladder.

There has been limited success to develop an efficacious UTI vaccine that would provide protection from infection by UPEC. Immunization with Solco-Urovac, a vaccine formulation that is comprised of inactivated uropathogenic bacteria, generates urinary IgA [48] and vaginal immunization with this vaccine provides protection that lasts 8–12 weeks in humans [11]. If administered more frequently, vaccination with Solco-Urovac can increase time to recurrence to six months [49]. This suggests this vaccine might be useful to prevent recurrent UTIs in susceptible populations. The major Type 1 fimbrial adhesin of UPEC, FimH, has also been used as a vaccine candidate and provided protection in mice [14] and prevented bacteriuria in primates [15]. One of the vaccine candidates we identified and tested, IroN, has been used previously to immunize mice, and similar to our results with IroN, was shown to generate antigen-specific IgG but not IgA [17]. In contrast to our findings, this group found that IroN protected mice against renal infection. This difference could be accounted for by the fact that we used an IroN extracellular loop-derived peptide mixed with CT and the previous study used denatured protein without adjuvant [17]. More recently, it has been shown that intranasal immunization with formalin-killed UPEC lacking capsular polysaccharides and O antigen generates a specific humoral response in mice; however, the protective efficacy of this vaccine is not known [50]. Lastly, using a lethal challenge model of extraintestinal pathogenic *E. coli* infection, it was found that vaccination with IroN, but not ChuA, increased survival in immunized mice [22].

Despite these advances, a UTI vaccine that confers long-term protection against uncomplicated UTIs is currently lacking. The rational large-scale screening process we described identified six surface-exposed outer membrane receptors for iron compounds. These represent vaccine candidates because they are conserved among uropathogenic strains and largely absent from commensal bacteria, induced during growth in human urine and during experimental UTI, and are antigenic. Iron acquisition is well known to be a common trait necessary for bacterial pathogenesis and by targeting receptors involved in this process it may be possible to disrupt this critical function in addition to pathogen neutralization and opsonization. Intranasal inoculation with three of six vaccine candidates (IreA, Hma, IutA) provided protection from challenge with uropathogenic *E. coli* and this study provides the basis for the development of a subunit vaccine that would incorporate these protective antigens to provide broader efficacy within the urinary tract and across uropathogenic isolates. Importantly, we have shown that focusing on an entire class of proteins that are involved in a singular function necessary for pathogenesis rather than a single protein may be fundamental strategy that could be generally adopted during the development of vaccines against pathogens.

## Materials and Methods

### Bacterial strains and culture conditions

*E. coli* strain CFT073 was isolated from the blood and urine of a patient with acute pyelonephritis [51]. *E. coli* strain 536 was isolated from a patient with acute pyelonephritis [52]. Unless otherwise noted, bacteria were cultured in Luria broth (LB) containing appropriate antibiotics (100 µg/ml ampicillin, 25 µg/ml kanamycin, and/or 20 µg/ml chloramphenicol) at 37°C with aeration.

### Murine model of ascending UTI

Female CBA/J mice were transurethrally inoculated as previously described [53]. Prior to inoculation, overnight *E. coli* CFT073 cultures were harvested by centrifugation (3000×*g*, 30 min 4°C) and resuspended in PBS to an OD_600_ of 4.0, equivalent to 4×10^9^ CFU/ml. Bacterial suspension (50 µl/mouse) was delivered transurethrally using a sterile 0.28 mm inner diameter polyethylene catheter connected to an infusion pump (Harvard Apparatus), with total inoculum of 1×10^8^ CFU/mouse. For determination of CFUs, organs were harvested from euthanized animals at 48 h post-inoculation and homogenized in PBS with a GLH homogenizer (Omni International). Bacteria in tissue homogenates were enumerated by plating on Luria-Bertani agar containing 0.5 g/L NaCl using an Autoplate 4000 spiral plater (Spiral Biotech). Colonies were enumerated using a QCount automated plate counter (Spiral Biotech).

Blood was collected as necessary from anesthetized mice by an infraorbital bleed using 1.1 to 1.2 mm Micro-Hematocrit Capillary Tubes (Fisher) and serum was separated using Microtainer Serum Separator Tubes (Becton Dickinson). Six- to eight-week old mice were used for these studies and animals were ≤15 weeks old at the conclusion of all experiments. All procedures were conducted according to protocols approved by University Committee on the Care and Use of Animals at the University of Michigan.

### Antigen purification

Genes encoding the selected antigens were PCR-amplified from CFT073 genomic DNA and cloned into either pBAD-*myc*-HisA (Invitrogen) or pET30b+ (Novagen). Recombinant protein expression from pBAD (Hma, IutA, ChuA) was induced in *E. coli* TOP10 cultured to OD_600_ = 0.8 by addition of L-arabinose to 100 µM for 4 h. Proteins expressed from pET (Iha, IreA) were over-expressed in *E. coli* BL21(DE3) pLysS cultured in Terrific broth (12 g/L tryptone, 24 g/L yeast extract, 2.3 g/L KH_2_PO_4_, 12.5 g/L K_2_HPO_4_, 4% glycerol) to OD_600_ = 1.0 at 37°C and induced overnight with 1 mM isopropyl β-D-1-thiogalactopyranoside (IPTG).

Induced cultures were harvested by centrifugation (8,000×*g*, 4°C, 10 min), resuspended in 10 mM HEPES, pH 7, and 100 U Benzonase nuclease (Sigma). Bacteria were lysed by two passages though a French pressure cell (20,000 psi) and the lysate was cleared by centrifugation (8,000×*g*, 4°C, 10 min). Bacterial membranes were pelleted from the cleared lysate by ultracentrifugation (112,000×*g*, 4°C, 30 min) and the membrane pellet resuspended in 5 ml 100 mM NaH_2_PO_4_, 10 mM Tris-HCl, 8 M urea, 1% ASB-14, pH 8.0. His_6_-tagged proteins were purified on nickel-nitriloacetic acid-agarose columns (Qiagen) under denaturing conditions according to the manufacturer's instructions (The QIAexpressionist). Eluted purified proteins were renatured by dialysis at 4°C into a final solution containing 0.05% Zwittergent in PBS, pH 7.5 and quantified using the BCA protein assay (Pierce).

### Peptide synthesis

Putative extracellular loops of IroN and IutA were predicted using the PRED-TMBB program (http://biophysics.biol.uoa.gr/PRED-TMBB/). 30-mer peptides corresponding loop 6 of IutA and loop 7 of IroN (IroN: YLLYSKGNGCPKDITSGGCYLIGNKDLDPE; IutA: VDDIDYTQQQKIAAGKAISADAIPGGSVD) were synthesized to ≥96% purity by Invitrogen.

### Vaccination

Purified antigens were chemically cross-linked to cholera toxin (CT) (Sigma) at a ratio of 10∶1 using *N*-succinimidyl 3-(2-pyridyldithio) propionate (SPDP) (Pierce) according to the manufacturer's recommendations. Peptide antigens were dissolved in 1 mM EDTA in PBS, mixed with reduced CT, and incubated at 4°C for 18 h. All immunizations were administered intranasally in a total volume of 20 µl/animal (10 µl/nare). Animals received a primary dose on day 0 of 100 µg crosslinked antigen (containing 10 µg CT) or 10 µg CT alone. Two boosts of 25 µg antigen (crosslinked to 2.5 µg CT) or 2.5 µg CT alone were given on days 7 and 14, and mice were challenged as described above.

### Tissue culture

Single-cell suspensions were made from spleens by forcing organs though 40 µm Cell Strainers (BD Falcon). Red blood cells were lysed for 2 min using 8.02 mg/ml NH_4_Cl, 0.84 mg/ml NaHCO_3_, 0.37 mg/ml EDTA in distilled water. Final suspensions were made in RPMI (supplemented with L-Glutamine, Gibco) with 1% sodium pyruvate, 1% L-glutamine, 1% penicillin/streptomycin, 1% non-essential amino acids, 10% fetal bovine serum (FBS), 0.001% 50 mM β-mercaptoethanol. Splenocytes (1.5×10^6^ cells/ml) were cultured with either medium alone or with 1 µg/ml purified antigen. After incubation of cells at 37°C, 5% CO_2_ for 48 h, supernatants were harvested and stored at −20°C.

### ELISA

For indirect serum ELISA, EIA/RIA medium binding 96-well ELISA plates (Corning) were coated at rt overnight with 5 µg/ml purified protein (for serum) or 10 µg/ml purified protein (for urine) diluted in carbonate buffer, pH 9.8. Non-specific binding sites were blocked with with 10% FBS, 0.04% NaN_3_ in PBS at room temp for 1 h. 1∶128 dilutions of mouse serum in blocking buffer or 50 µl of pooled undiluted urine were applied to wells in two to five replicates. Goat anti-mouse IgA, IgG, and IgM were obtained conjugated to alkaline phosphatase (Southern Biotech). Alkaline phosphatase substrate, *p*-nitrophenyl phosphate (1 mg/ml) (Sigma), was diluted in carbonate buffer and applied to wells at rt until color developed. The reaction was stopped with NaOH and read with a μQuant plate reader (Bio-Tek Instruments, Inc.) at a wavelength of 405 nm.

For cytokine ELISA, purified IL-17A, IFN-γ, and their corresponding matched antibody pairs were obtained from R&D Systems and used according to the manufacturer's recommendations. For detection, OPD Easy-tablets (2 mg/tablet, Acros Organics) were diluted and applied until color developed. The reaction was stopped by addition of 100 µl 6 N H_2_SO_4_ and read with a plate reader at a wavelength of 490 nm. Plates were washed by flooding all wells four times with wash buffer between all incubations.

### Statistical analysis

All graphing and statistical analyses were done using GraphPad Prism 5. Significance was determined using Mann-Whitney tests. Correlates of protection were determined using the Pearson correlation coefficient with linear regression to generate a best fit line. All statistics were conducted using 95% confidence intervals where applicable.

## Supporting Information

Figure S1 Circular dichroism spectrum of purified renatured Hma. Far UV CD spectral analysis of purified Hma (800 µg/ml) following buffer exchange and renaturation. Spectrum was measured from 195 to 250 nm at 25°C using a Jasco Co. (Tokyo, Japan) J-810 Rev. 1.00 instrument.
(0.04 MB PDF)
