# Supplementary figures and images for: Mucosal Immunization with Iron Receptor Antigens Protects against Urinary Tract Infection
Source: PLoS Pathog. 2009 Sep 18;5(9):e1000586. doi: 10.1371/journal.ppat.1000586 (PMC2736566; doi:10.1371/journal.ppat.1000586)

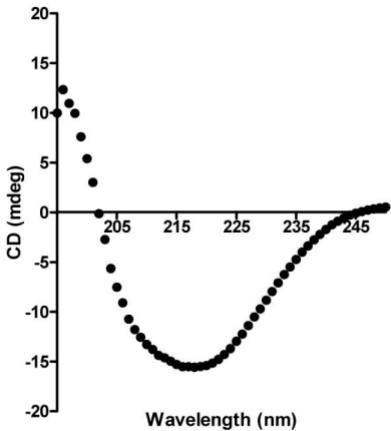

Supplement: Figure S1 — Circular dichroism spectrum of purified renatured Hma. Far UV CD spectral analysis of purified Hma (800 µg/ml) following buffer exchange and renaturation. Spectrum was measured from 195 to 250 nm at 25°C using a Jasco Co. (Tokyo, Japan) J-810 Rev. 1.00 instrument. (0.04 MB PDF) [file ppat.1000586.s001.pdf]
